# From Radical Treatment to Structured Active Surveillance: A Narrative Review of Low-Risk Localized Prostate Cancer

**DOI:** 10.3390/jcm15218290

**Published:** 2026-10-24

**Authors:** Patricia Rodríguez-Parras, Pedro Román-Ramos, Alberto Zambudio-Munuera, Miguel Ángel Arrabal-Polo

**Affiliations:** 1 Department of Urology, Hospital Universitario Clínico San Cecilio, Avenida del Conocimiento s/n, 18016 Granada, Spain; 2 Group MP-18, Institute of Biosanitary Research of Granada (ibs.GRANADA), 18012 Granada, Spain; 3 Department of Surgery, University of Granada, 18071 Granada, Spain

**Keywords:** prostatic neoplasms, active surveillance, low-risk prostate cancer, magnetic resonance imaging, genomic classifiers, watchful waiting

## Abstract

Active surveillance (AS) is the preferred initial strategy for suitable men with confirmed low-risk localized prostate cancer and sufficient life expectancy to benefit from future curative treatment. The objective of this review is to determine which components of contemporary surveillance practice are supported by trial and cohort evidence, which remain adjunctive or investigational, and to integrate this appraisal into an explicit, life-expectancy-aware decision pathway. This narrative review traces the transition from radical treatment through historical conservative management trials to contemporary surveillance. The original search covered literature up to 10 September 2026; supplementary retrieval and verification were performed on 10 October 2026. Evidence is interpreted by population, design and endpoint. Watchful waiting, PIVOT observation and ProtecT active monitoring are distinguished from structured AS. Clinical selection, the exclusion of cribriform/intraductal carcinoma, MRI-informed sampling and protocol-based reassessment underpin surveillance. Stable MRI permits selected biopsy individualization under EAU guidance but does not universally replace histological reassessment. Tissue classifiers and germline findings have heterogeneous prognostic evidence; the reviewed studies do not establish improved outcomes from molecularly guided AS. Selected MRI-based strategies were economically favorable within US models, without demonstrating biopsy-omission safety. Psychological outcomes and reasons for changing treatment vary. Focal ablation remains investigational. An author-proposed, unvalidated pathway separates PSA/MRI concern from confirmed clinically relevant reclassification and includes transition to watchful waiting when health or life expectancy changes.

## 1. Introduction

The management of low-risk localized prostate cancer has shifted toward avoiding treatment morbidity while preserving the opportunity for cure. Longitudinal surveillance cohorts, rather than population incidence statistics, support the favorable prognosis of appropriately selected disease. In Canary PASS, 90% of 2155 participants had grade group (GG) 1 cancer; estimated 10-year incidences of metastasis and prostate cancer death were 1.4% and 0.1%, respectively [1]. These outcomes apply to a selected, monitored cohort and do not establish that every biopsy diagnosis of GG1 is low risk.

Conservative management terminology matters. Watchful waiting (WW) generally anticipates symptom-directed, palliative treatment when curative benefit is unlikely. AS retains curative intent through planned reassessment and selective delayed treatment. PIVOT used observation, whereas ProtecT used predominantly PSA-triggered active monitoring; neither intervention is identical to contemporary MRI-informed AS. Conflating these approaches can exaggerate the apparent uncertainty surrounding modern surveillance. Chronological comparison also requires attention to changing diagnostic intensity, grade interpretation and competing mortality [2,3,4].

The scope is localized low-risk cancer defined by grade, PSA and clinical stage, not GG1 alone. EAU 2026 classifies low risk as GG1, PSA < 10 ng/mL and cT1–2 assessed by digital rectal examination (DRE). Many classic surveillance cohorts instead used cT1c–T2a, PSA ≤ 10 ng/mL and additional volume criteria. PSA density (PSAD), MRI visibility and biopsy tumor burden refine selection but should not be substituted for the stated risk definition [4,5,6]. The historic very-low-risk category commonly combined cT1c, GG1, PSA < 10 ng/mL, PSAD < 0.15 ng/mL/cm^3^, fewer than three positive cores and ≤50% cancer per core; NCCN Version 5.2026 has eliminated this separate category [4,7]. Historical cohort eligibility remains relevant even when current guideline labels change.

Evolutionary accounts and surveillance frameworks already exist, including the perspective of Moore and Parker, the prostate-and-thyroid review of Lowenstein and colleagues, Klotz’s contemporary review and the 2026 risk-adapted framework of Zattoni and colleagues [8,9,10,11]. Building on but distinct from these accounts, the objective of this review is threefold: (i) to interpret historical and contemporary surveillance evidence by population, trial design and clinical endpoint rather than by label alone (Section 3 and Section 4); (ii) to appraise the incremental, rather than purely associative, value of MRI-based follow-up, tissue classifiers and germline findings for AS selection and monitoring (Section 4 and Section 5); and (iii) to integrate this appraisal, together with the economic, access and psychological evidence reviewed in Section 6, into an explicit, life-expectancy-aware decision pathway that separates PSA/MRI concern from confirmed clinically relevant reclassification (Section 9). Patient concerns are considered throughout without assuming a uniform psychological penalty from surveillance.

## 2. Methods

### 2.1. Narrative Design and Original Search

We conducted an evidence-based narrative review using PubMed/MEDLINE, Scopus and reference-list checking for literature available up to 10 September 2026. Search concepts included prostate cancer, AS, WW, MRI, genomic classifiers and focal therapy. Three reviewers selected studies for explanatory value and resolved disagreements through discussion; synthesis was organized by clinical era and theme. Selection was purposive rather than exhaustive and did not use independent duplicate screening. Complete database-specific strings, retrieval counts and filter settings for this initial search were not retained in the documented search summary. The reproducible supplementary retrieval described below is therefore reported separately. No registered protocol, formal risk-of-bias instrument, PRISMA flow diagram or quantitative pooling was used.

### 2.2. Supplementary Retrieval and Source Verification

A supplementary PubMed search was executed on 10 October 2026, with a publication-date upper bound of that day and no language or publication-type filters. A broad AS/WW/active-monitoring query returned 5030 records. Six additional topic queries addressed imaging, molecular classifiers, germline findings, economics, psychology/access and focal treatment. Appendix A provides their exact submitted strings and recorded counts. These are overlapping retrieval counts obtained with retmax = 0, not numbers of screened or included studies; the broad and topic queries were not exhaustively screened. The supplementary work prioritized reviewer-nominated primary studies, landmark trials/cohorts, contradictory molecular and germline findings, and guideline/registry verification. Citation chasing and targeted DOI/PMID checks complemented these searches. This new strategy is not presented as the original September search.

Bibliographic records and published abstracts were checked against indexed primary records; available original full texts were consulted for disputed methods and results, including the economic studies by Kang and Magnani. EAU 2026 full guidance and ClinicalTrials.gov records were inspected. Appendix B identifies full-text, abstract-only and bibliographic-only access by reference. The published abstracts of NCCN Version 5.2026 and the AUA/ASTRO 2026 amendment were available, but their complete recommendations were not accessible during this revision. Detailed Decipher version comparison methods and the 10-year numbers at risk in the 354-person MRI cohort were also unavailable. These access limits are identified at the relevant passages, unverified details are omitted and older schedules are not represented as verified 2026 recommendations [4,7,12,13,14,15,16].

### 2.3. Appraisal and Synthesis

Evidence was prioritized according to the question. Randomized trials inform comparative treatment effects and the diagnostic pathway; their risk mix, treatment era, crossover and endpoint timing constrain transfer to modern GG1 AS. Prospective longitudinal cohorts inform absolute surveillance outcomes, with attention to entry selection, duration, numbers at risk, censoring and competing death. Registry/claims analyses inform adoption and access but may not ascertain treatment intent or monitoring quality. For imaging, randomized biopsy studies and diagnostic meta-analysis take precedence over narrative summaries, while reference standards and verification bias remain relevant. Molecular studies are evaluated for analyzed denominators, missing tissue, endpoint definition, adjustment for clinical predictors, external validation and whether test-guided care improves outcomes. Nonrandomized treatment comparisons are assessed for confounding and treated volume. Economic models are interpreted through population, perspective, costs, assumptions and sensitivity rather than as clinical safety trials.

Study selection remains purposive and is not exhaustive. Both favorable and negative findings are retained when they change the clinical interpretation. SANRA was used for a separate self-assessment of narrative review quality, not as a risk-of-bias tool or a substitute for systematic review methods [17]. Table 1 summarizes the documented process and its limits. The completed self-assessment is provided in Supplementary Material S1.

### 2.4. Generative Artificial Intelligence

During manuscript preparation, Claude Sonnet 5.5 (Anthropic) was used for literature retrieval support, language editing, structural organization and reference consistency checking. GenAI outputs were treated as drafting aids: scientific statements and citations were checked against the indexed primary records, available full texts, official guidance and trial registrations used in this review. Source access limitations are stated in Section 2.2 and Appendix B. GenAI was not used to generate patient data or empirical images. The authors have reviewed and edited the output and retain full responsibility for the originality, validity, integrity and final content of the manuscript.

## 3. Historical Evidence and Contemporary Surveillance

### 3.1. Radical Treatment Versus Earlier Conservative Care

SPCG-4 randomized 695 men with predominantly clinically detected localized cancer to radical prostatectomy or WW in a largely prescreening setting. With a follow-up of up to 29 years, 71 of 347 surgery-assigned men and 110 of 348 WW-assigned men died from prostate cancer; the reported relative risk was 0.55 (95% CI 0.41–0.74) [18]. The counts are crude events across prolonged follow-up, not fixed-time cumulative incidence estimates. The trial establishes benefit from surgery in its population, with substantial competing mortality and stronger relevance to clinically evident disease than to today’s MRI-selected low-risk cohort.

PIVOT randomized 731 men in the early PSA era to surgery or observation. Extended follow-up reached 22.1 years (median 18.6 years among survivors); all-cause mortality was 68% versus 73% (HR 0.84, 95% CI 0.70–1.00). The absolute difference was 5.7 percentage points (95% CI −0.89 to 12) [2]. Risk-stratified estimates suggested smaller absolute benefit in low-risk disease, but subgroup uncertainty and a risk-mixed population limit conclusions about an individual GG1 patient. Observation was not a modern MRI/biopsy AS protocol. Neither SPCG-4 nor PIVOT justifies grouping WW, observation and AS as a single comparator.

### 3.2. ProtecT and the Meaning of Active Monitoring

ProtecT randomized 1643 PSA-detected cases to active monitoring (*n* = 545), prostatectomy (*n* = 553) or radiotherapy (*n* = 545). At median 15-year follow-up, prostate cancer deaths were 17 (3.1%), 12 (2.2%) and 16 (2.9%), respectively (*p* = 0.53), while metastases occurred in 51 (9.4%), 26 (4.7%) and 27 (5.0%) [3]. Clinical progression was 25.9%, 10.5% and 11.0%. Active monitoring therefore had more metastatic/progression events despite similarly low disease-specific mortality; the absence of a mortality difference is not proof of equivalence for every endpoint. Updated characterization placed over one-third of participants in intermediate/high-risk groups. Baseline assessment lacked the contemporary MRI-informed pathway, and monitoring relied mainly on PSA, so these estimates cannot be transferred directly to confirmed low-risk AS. Randomized patient-reported outcomes document distinct urinary, sexual and bowel effects of treatment, which should inform shared treatment decisions [19].

### 3.3. Structured Surveillance Cohorts and Adoption

PRIAS operationalized eligibility using clinical T1/T2, PSA ≤ 10 ng/mL, Gleason score ≤ 6, one or two positive cores and PSAD < 0.2 ng/mL/cm^3^. The initial 2494-person report used PSA every 3–6 months and repeat biopsies at years 1, 4 and 7. Median follow-up was only 1.6 years; reclassification occurred in 415 of 1480 rebiopsied men and two-year treatment-free survival was 77.3% [5]. Its 100% disease-specific survival should not be interpreted as long-term proof of safety. The Toronto cohort included favorable intermediate-risk as well as low-risk disease: among 993 men, median follow-up among survivors was 6.4 years and estimated 10-/15-year cancer-specific survival was 98.1%/94.3% [20]. These eligibility differences explain why cross-cohort outcome rankings are unreliable.

Canary PASS provides stronger contemporary long-term context. This prospective, multicenter cohort enrolled 2155 men, 90% with GG1, and used protocol-directed testing and repeat biopsy. Median follow-up was 7.2 years. The estimated 10-year incidences were 43% for biopsy grade reclassification (95% CI 40–45), 49% for treatment (47–52), 1.4% for metastasis (0.7–2.0) and 0.1% for prostate cancer death (0–0.4). There were 21 metastatic events and three prostate cancer deaths during observed follow-up [1]. Thus, treatment and reclassification are much more frequent than lethal progression. These cohort results support structured AS while leaving selection effects, evolving diagnostic practice and limited late-event counts relevant.

Population-based data add a counterpoint. In an Ontario low-grade cohort of 21,282 men, with a median follow-up of 9.8 years, the AS group had 10-year metastasis-free survival of 94.2% and cancer-specific survival of 98.1%. Compared with initial treatment, adjusted hazards were higher for metastasis (HR 1.34, 95% CI 1.15–1.57) and prostate cancer mortality (HR 1.66, 1.15–2.39), with an approximately one-percentage-point cancer-specific survival difference [21]. Administrative assignment, a 24-month landmark and residual confounding constrain causal interpretation. These findings neither negate selected protocol-based cohorts nor permit a claim of identical long-term outcomes across all real-world surveillance.

Adoption is distinct from oncological outcome. Tzeng and colleagues analyzed 137,915 NCDB low-risk diagnoses: deferred treatment increased from 14.7% in 2010 to 46.3% in 2017. In multivariable analyses, Black men, lower-income men and men with governmental insurance or no insurance were more likely to receive deferred treatment, but experienced smaller increases over time [22]. The NCDB variable combines AS and WW and does not establish either intent or adherence. AQUA provides more recent practice data: among 20,809 low-risk cases from 349 practices and 1945 clinicians, AS rose from 26.5% in 2014 to 59.6% in 2021, with practice-level rates of 4.0–78.0% [6]. Automated electronic record ascertainment and substantial missing race data limit explanations of these differences.

Claims-based monitoring intensity also requires caution. In older SEER–Medicare men initially managed without active treatment, only 39% initiated claims-defined AS monitoring during the first year [23]. A low testing frequency may reflect access barriers, incomplete ascertainment, nonadherence, individualized care or WW; it does not itself demonstrate a palliative treatment intention. Treatment-intent terminology should therefore follow documented clinical goals rather than claims alone.

## 4. MRI, Biopsy and Guideline-Based Follow-Up

### 4.1. Initial Assessment and Complementary Sampling

Because both multiparametric and biparametric protocols are used for active surveillance, MRI is used throughout this review as the general term; acquisition type is not distinguished unless a cited study’s findings specifically depend on it. MRI informs lesion localization, baseline visibility and sampling but cannot verify whole-gland grade by itself. In ASIST, 273 men with newly diagnosed GG1 were randomized to systematic confirmatory biopsy or MRI with targeted plus systematic sampling. Among those undergoing confirmatory biopsy, centrally reviewed upgrading occurred in 42/127 (33%) in the MRI arm and 36/132 (27%) in the non-MRI arm (*p* = 0.3) [24]. At two years, among the 199 men who continued AS after confirmation, AS failure was 19/98 (19%) versus 35/101 (35%; *p* = 0.017) [25]. Failure included grade/clinical progression, patient choice, clinical judgment, treatment or loss to follow-up. The initial diagnostic endpoint and later composite therefore address different questions, and the conditional follow-up population and center effects require attention.

Targeted and systematic biopsies detect complementary reclassification. In Yerram and colleagues’ single-center cohort, eligibility included any-volume GG1 and selected GG2. Among 277 men sampled at two years, combined biopsy identified 90 grade progressions, compared with 73 using targeted and 44 using systematic biopsy. Targeted biopsy alone detected 46/90 (51.1%), whereas systematic biopsy alone detected 17/90 (18.9%; 95% CI 12.1–28.2) [26]. The study supports appropriate combined assessment, not abandoning systematic sampling universally or extrapolating mixed-grade yields to a pure low-risk cohort.

### 4.2. Serial MRI and Observed Outcomes

Serial MRI standardizes longitudinal review but has limited sensitivity for histological progression. A meta-analysis of 15 studies and 2240 men reported pooled sensitivity 0.59 (95% CI 0.44–0.73) and specificity 0.75 (0.66–0.84). Performance did not differ significantly between PRECISE and institutional progression definitions (*p* = 0.37 for sensitivity and *p* = 0.74 for specificity) [27]. Heterogeneous entry criteria, MRI thresholds and biopsy reference standards limit transportability. PRECISE version 2 improves reporting, including separate stable categories for visible/nonvisible lesions and an image-quality assessment, but is expert consensus rather than proof that serial imaging can replace repeat biopsy [28].

Baseline MRI visibility is prognostic information rather than a stand-alone treatment trigger. In the 1150-person MRI-led cohort, 36% had GG2 and 55% an MRI-visible lesion. At the median follow-up of 64 months, five-year event-free survival in GG1 was 91% (95% CI 88–94) for nonvisible versus 71% (65–78) for visible disease; the endpoint was upgrade to Gleason ≥4+3 or transition to treatment. Only 487 participants had follow-up biopsy, illustrating selective verification, and ten metastatic events were reported [29]. Selection, discretionary sampling and treatment decisions prevent this association from proving the safety of universal biopsy omission.

The separate 354-person GG1 cohort reported a median follow-up of 6.3 years (IQR 3.2–9.1), two metastatic events and no prostate cancer deaths [16]. Its abstract described a 0.5% ten-year cumulative incidence of metastasis; that is not the crude fraction 2/354. Because the full curve and ten-year numbers at risk were unavailable, the late percentage and estimated ten-year AS-retention rate are omitted from Table 2. These favorable observed outcomes cannot establish that MRI caused a survival improvement over earlier cohorts.

### 4.3. Practical Surveillance and Differences Between Current Guidance

EAU 2026 states that “A stable MRI (PRECISE 1–3) does not make repeat biopsy superfluous”, while its recommendation permits repeat-biopsy omission in selected low-risk patients with stable MRI (PRECISE 3) and stable PSAD < 0.15 ng/mL/cm^3^ when MRI is repeated before biopsy (weak recommendation). The general protocol specifies PSA at least every six months, DRE, and repeat biopsy every 2–3 years until life expectancy is below ten years (strong recommendation). MRI does not provide an unconditional exemption from sampling [4].

Confirmatory assessment depends on the diagnostic pathway. EAU advises MRI before confirmatory biopsy if it was not performed initially (strong), and targeted/perilesional cores from PI-RADS ≥ 3 lesions at confirmation or repeat biopsy (strong). A separate confirmatory biopsy may be omitted after upfront MRI with targeted and perilesional sampling (weak); confirmation remains particularly relevant when initial sampling or imaging was incomplete. Follow-up includes PSA at least six-monthly, clinical/health review and repeat biopsy as above; annual DRE is the consensus default, with potential de-escalation during regular MRI surveillance. EAU does not impose a universal fixed MRI interval: MRI and repeat biopsy are recommended for rising PSA with a doubling time < 3 years (strong). MRI progression also requires sampling before a treatment change. Treatment decisions should rest on biopsy-confirmed progression rather than PSA/MRI changes alone (weak) [4].

An apparent upgrade may reveal higher-grade tumors missed at baseline rather than newly evolved disease. Clinical review should check PSA variability, PSAD, MRI quality/visibility, adequacy of prior sampling and pathology before defining clinically relevant overall risk reclassification. A finding of limited GG2 does not automatically mandate immediate treatment: its eligibility belongs to the separate selected intermediate-risk discussion in Section 7. Table 3 distinguishes verified EAU recommendations from the accessible 2026 NCCN/AUA summaries, preserving rather than concealing the incomplete cross-guideline comparison.

## 5. Molecular and Hereditary Information

### 5.1. Tissue Classifiers and Clinical Utility

A prognostic association is a relationship between a test and an outcome; incremental prediction asks whether the test improves performance beyond established clinical factors; clinical utility asks whether using it changes decisions in a way that improves patient outcomes. These are separate claims. EAU regards tissue classifiers as promising adjuncts whose place alongside MRI requires further evidence. The analyses below do not demonstrate that integrating every molecular test into AS produces safer deferred treatment [4].

The Decipher version study analyzed 1579 men, including 605 on AS and 974 undergoing prostatectomy. Its AS endpoint was major upgrading to GG ≥ 3. Biopsy scores were lower with version 1.1 than 1.0 (median 0.34 versus 0.45); a high version 1.1 score (>0.6 versus <0.4) was associated with upgrading (HR 2.11, 95% CI 1.14–3.89), while version 1.0 did not show that association in the abstract [13]. Significance for one version and nonsignificance for another do not establish a statistically significant performance difference. AS follow-up, adjustment details and a direct discrimination/calibration comparison could not be checked in the accessible abstract, so neither adjusted superiority nor improved clinical utility is asserted. The two versions’ different score distributions also caution against assuming that historical cutoffs are interchangeable.

Negative external cohort evidence is important. In Canary PASS, commercial 17-gene GPS was available in 432 men, with a median follow-up of 4.6 years. There were 167 biopsy upgrades and 101 subsequent prostatectomies, of which 52 had adverse pathology. GPS was not associated with upgrading (*p* = 0.48). Its adverse pathology association was no longer statistically significant after including PSAD with diagnostic grade (*p* = 0.066), and it did not significantly improve stratification beyond those clinical factors [30]. This independent cohort assessment limits a general claim that genomic classifiers always add actionable information, without proving that no selected patient can benefit.

MAST molecular analyses were internal studies of a single-center prospective protocol that enrolled 205 low/favorable intermediate-risk men with MRI and annual biopsies for up to three years. In the PAM50 analysis, 159 men had successful profiling of 410 cores, but only 128 had baseline transcriptome data: 46 Luminal A, 26 Luminal B and 56 Basal. Attrition primarily reflected unavailable outside diagnostic tissue, negative confirmation or insufficient tumor, with fewer RNA quality failures. Luminal B had shorter unadjusted grade-progression-free survival (median 1.7 versus 2.9 years; log-rank *p* = 0.005), but did not retain a significant association after adjustment for age, MRI visibility, PSAD and positive-core count. Progression meant GG1 to GG ≥ 2 or GG2 to GG ≥ 3. Median follow-up among the 63 nonprogressors was 1.86 years; 27 reached at least two years [31]. Small subgroups, missing tissue, internal analysis and industry co-authorship limit the claim; unadjusted subtype separation is not established incremental utility.

The companion MAST analysis profiled 141/205 men, comprising 324 GG1 cores from 235 biopsy sessions. Across all analyzed GG1 cores, derived GPS was higher when GG ≥ 3 coexisted elsewhere (median 0.24 versus 0.10), but none of the signatures distinguished coexisting GG ≥ 2. With the highest-volume GG1 core per biopsy, which approximates clinical test selection, no signature showed a group difference. Mixed-model adjustment for age, PSAD and PI-RADS did not establish independent prediction [32]. GG ≥ 3 was present alongside only nine of the analyzed cores. Scores called dGPS and dCCP were retrained microarray research signatures, not the proprietary commercial RT-PCR algorithms; their results cannot be assumed to validate commercial GPS or Prolaris. Insufficient/archival tissue and industry-linked coauthors further constrain generalizability. This endpoint concerns occult higher grade in the same biopsy session, not subsequent metastatic progression.

G-MAJOR (NCT04396808) tests a more direct management question using a cluster-crossover randomized design with quarter-year assignment periods. The registry lists actual enrollment of 805 men and a primary endpoint of remaining on AS without treatment at two years. On 10 October 2026 it was active, not recruiting, with estimated primary completion in March 2028 and no posted results [33]. Its design may address the effect of Decipher-guided management, but a registered objective is not evidence of benefit.

### 5.2. Germline Findings by Gene and Clinical Setting

Inherited susceptibility, screening of unaffected carriers and prognosis after an AS diagnosis must be distinguished. Carter and colleagues sequenced 1211 men from two AS cohorts; median follow-up of the analyzed population was three years. Grade reclassification occurred in 11/26 carriers of pathogenic ATM/BRCA1/BRCA2 variants versus 278/1185 noncarriers (adjusted HR 1.96, 95% CI 1.004–3.84). For BRCA2, 6/11 carriers were reclassified (adjusted HR 2.74, 1.26–5.96). Competing risk analyses adjusted for age, PSAD, cancer-positive core count and genetic background [34]. These small carrier numbers and a biopsy-grade endpoint support caution, especially for BRCA2, but do not establish a gene-specific mortality benefit from immediate treatment.

Brady and colleagues examined 437 Canary PASS participants in a nested case–control analysis with a median follow-up of 7.2 years. Only eight carried pathogenic BRCA1/BRCA2/ATM variants, including three BRCA2 carriers. Carrier prevalence was similar in men with and without grade reclassification (1.9% versus 1.8%). For the broader DNA-repair-gene set, the adjusted association with adverse characteristics was also nonsignificant (HR 0.73, 95% CI 0.29–1.86); this composite association should not be mislabeled as a grade-reclassification hazard [35]. The negative findings temper certainty but do not provide precise reassurance for BRCA2 because carriers were so few. Evidence for BRCA1, ATM, HOXB13 and mismatch repair genes should not be inferred from the BRCA2 estimate or from inherited susceptibility alone.

Testing decisions follow personal/family history and guideline indications rather than routine repeat testing during AS. EAU recommends testing with a known familial high-risk mutation or multiple relevant cancers on the same side of the family (strong), advises consideration with multiple relatives diagnosed with prostate cancer before 60 years or a relative dying from prostate cancer before 60 (weak), and recommends germline testing when a BRCA mutation is detected somatically (strong), with genetic counseling. An already-known pathogenic BRCA2 result warrants individualized discussion of uncertainty, diagnostic adequacy and closer surveillance; it is not an automatic exclusion from AS when tumor characteristics are favorable under EAU discussion [4]. A variant of uncertain significance should not determine intervention, consistent with ACMG/AMP interpretation guidance [36]. Germline status is a baseline attribute, while health, tumor assessment and preferences are reviewed over time.

PATROL enrolls unaffected men at inherited risk; its primary endpoint is the positive predictive value of age-specific PSA thresholds with MRI for clinically significant cancer. Postdiagnosis AS follow-up is a secondary aim, so the protocol cannot establish current AS safety in carriers [37]. The SOGUG expert panel discussed measures to improve AS adherence and, separately, genetic testing to refine focal therapy indications; it did not demonstrate that testing improves surveillance adherence [38]. Neither a screening protocol nor expert opinion replaces direct carrier outcome evidence. Table 4 separates molecular and hereditary associations, negative findings and unresolved clinical utility.

## 6. Economics, Access and Patient Experience

### 6.1. Conditional Economic Findings

MRI-triaged strategies can be economically attractive under model-specific assumptions. Kang and colleagues simulated 60-year-old men with low-risk disease over a lifetime horizon, comparing WW, PSA plus annual biopsy without MRI, and MRI strategies with different schedules and PI-RADS thresholds. Diagnostic, treatment and visit costs were based on Medicare reimbursements or converted to 2019 US dollars; costs and QALYs were discounted by 3% annually. At a US$100,000/QALY threshold, annual MRI with biopsy at PI-RADS ≥ 4 yielded an incremental cost-effectiveness ratio of US$67,221/QALY. The 42% biopsy reduction specifically compared the ≥4 and ≥3 thresholds at annual MRI. Annual MRI strategies yielded 16.19 QALYs versus 16.14 without MRI and 15.94 with WW. Age, initial grade misclassification, subsequent treatment, adherence and procedure-related utilities affected the results [14]. The costs describe a US medical care setting; they do not establish a societal perspective or transferability to other health systems. These are model outputs, not observed outcomes or a validated biopsy omission rule.

Magnani and colleagues modeled GG1/cT ≤ 2 surveillance using PASS-derived risks and a US healthcare system perspective, Medicare reimbursement-based costs inflated to 2020 dollars, a lifetime horizon and 3% annual discounting. Comparators included immediate prostatectomy/radiotherapy, scheduled transrectal biopsy and MRI pathways with biopsy regardless of findings or only after positive MRI (PI-RADS ≥ 3). A biennial MRI-triaged strategy had an incremental cost-effectiveness ratio of approximately US$44,600/QALY at a US$50,000/QALY threshold. Its probability of being cost-effective was 48.3% at that threshold and 54.7% at US$100,000/QALY [15]. Assumed adherence, grade misclassification, MRI/biopsy accuracy, testing costs, treatment allocation and diagnostic-versus-surveillance MRI performance materially affected the preferred strategy.

The two models differ in population inputs, imaging thresholds, comparators and costs. Their estimates do not supply universal clinical PI-RADS cutoffs or MRI intervals and do not demonstrate the oncological safety of omitting biopsies. Missed higher grade, biopsy burden, infection/other complications and local access costs must be considered together. EAU’s selected stable-MRI/low-PSAD exception is a clinical recommendation with weak strength; it should not be expanded solely because a simulated biopsy-sparing strategy appears favorable. Transfer to other health systems requires local costs and pathway performance.

### 6.2. Access and Diagnostic Equity

Implementation requires access to reliable imaging, pathology, appropriate biopsy and longitudinal care. The NCDB and AQUA findings in Section 3 describe uptake rather than equivalent monitoring quality. Patel and colleagues’ race-related comparison concerned cancer detection using MRI-fusion-targeted diagnostic biopsy [39]; it is diagnostic evidence, not direct evidence of racial differences in AS metastasis, survival or adherence. Neither diagnostic detection differences nor administrative treatment labels alone identify the mechanism of unequal surveillance outcomes.

### 6.3. Psychological Outcomes and Reasons for Changing Treatment

Measured anxiety is different from patient concern, physician recommendation, treatment preference or loss to follow-up. Carter and colleagues’ systematic review included 34 studies and 12,497 participants; only five studies were rated high quality and 14 included WW or no active treatment rather than a clearly defined modern AS protocol. The review did not find a consistent adverse psychological effect of conservative monitoring compared with active treatment, but heterogeneity limits a universal reassurance claim [40]. Simpkin and colleagues synthesized 26 cohorts (7627 men; median follow-up 3.5 years), estimating an annual treatment change rate of 8.8% (95% CI 6.7–11.0). Biopsy findings, PSA triggers and anxiety-associated patient choice all contributed; that overall rate is not the proportion stopping AS because of anxiety [41].

HAROW followed only 52 intermediate-risk AS patients for a median 6.8 years; 36% of discontinuations were attributed jointly to patient or physician concerns [42]. This category was not a validated anxiety measurement and should not be extrapolated to all GG1 AS. The Finnish PRIAS study assessed low-risk quality of life over one year [43]; comparing it directly with HAROW cannot establish a risk-group difference in distress. Support should therefore address the individual patient’s understanding, measured symptoms, preferences and practical barriers, while distinguishing elective treatment from disengagement from follow-up.

## 7. Scope of Treatment Decisions and Selected GG2 Surveillance

For low-risk men with life expectancy > 10 years who remain eligible for future curative treatment, EAU recommends AS; for life expectancy < 10 years, WW is recommended (both strong). Frailty, comorbidity, competing mortality and functional priorities matter more than chronological age alone. At borderline life expectancy, decisions require individualized assessment. Transition from AS to WW when curative treatment would no longer offer meaningful benefit is a change in therapeutic intent, not poor adherence [4].

Selected GG2 is outside the principal low-risk algorithm. EAU permits AS in carefully chosen GG2 disease, for example <10% Gleason pattern 4, PSA < 10 ng/mL, ≤cT2a, limited MRI extent, ≤3 GG2-positive cores and ≤50% involvement per core, with acceptance of a higher metastatic progression risk (weak). Cribriform or intraductal carcinoma excludes AS (strong); pathology review is particularly important when limited pattern 4 is reported. This exception does not make all favorable intermediate-risk disease equivalent to low risk. Risk-mixed evidence from Toronto, Yerram, MAST and MRI-led cohorts is identified explicitly [4].

Confirmed clinically meaningful reclassification should prompt reassessment of cancer risk, curative benefit and preferences. Prostatectomy or radiotherapy may then be discussed as appropriate to the revised risk category. Imaging or PSA concern alone should first prompt review and appropriate sampling; small-volume GG2 may still meet the separate selected-AS criteria. Randomized ProtecT patient-reported data illustrate the competing urinary/sexual effects of surgery and bowel/sexual effects of radiotherapy [19]. Treatment choices should reflect absolute disease risk and patient priorities rather than an automatic reaction to a single test.

## 8. Focal and Other Ablative Treatment

Lesion-directed focal ablation, subtotal ablation and whole-gland treatment are distinct interventions. Focal therapy is not the routine next step for stable low-risk men and has not shown a survival advantage over contemporary AS in the evidence reviewed. EAU confines focal HIFU/cryotherapy to a prospective registry and other focal modalities to well-designed prospective trials; for low-risk disease, its treatment discussion emphasizes avoiding overtreatment [4].

PCM301 randomized 413 low-risk men to vascular-targeted photodynamic therapy (*n* = 206) or AS (*n* = 207). At 24 months, progression occurred in 58/206 (28%) versus 120/207 (58%; adjusted HR 0.34, 95% CI 0.24–0.46), and negative biopsy was more frequent after ablation (49% versus 14%) [44]. These short-term biopsy outcomes reflect an earlier surveillance protocol and do not demonstrate reduced metastasis or mortality relative to contemporary MRI-informed AS.

HIFI was a nonrandomized French comparison of whole-gland or subtotal HIFU (*n* = 1967) with prostatectomy (*n* = 1361). Intermediate-risk disease represented 61% and 64% of the groups; median ages differed substantially (74.7 versus 65.1 years). The 30-month salvage-treatment-free survival comparison (HR 0.71, 95% CI 0.52–0.97) supported the prespecified noninferiority analysis but is vulnerable to treatment selection confounding [45]. It neither establishes lesion-directed focal efficacy nor superiority over AS. PART randomized 82 men with unilateral intermediate-risk disease to partial HIFU or surgery to test recruitment feasibility, not comparative oncological efficacy [46].

IRE series also predominantly concern higher-risk disease: the 229-person five-year cohort contained 86% intermediate- and 7% high-risk cancers, with a median follow-up of 60 months and a five-year failure-free survival of 84% [47]. Prospective focal cryotherapy studied clinically significant cancer rather than a pure GG1 AS population [48]. Such studies describe treatment performance in their own setting and cannot validate the routine ablation of stable low-risk disease. The pooled sexual function literature is heterogeneous in baseline function, ablation extent, follow-up and hormonal exposure, so pooled IIEF-5 values should not rank modalities by preservation of function [49].

The directly relevant phase 3 HIFUSA study (NCT03531099) randomizes low-risk men to focal HIFU or AS. The registry reports actual enrollment of 108 and a primary endpoint of conversion to radical treatment at 48 months. At access on 10 October 2026, it remained active, not recruiting, with an estimated primary completion of 2 October 2026 and no posted results [50]. A past estimated completion date does not establish completion or efficacy. After ablation, follow-up must assess treated and untreated tissue with PSA, MRI and appropriate biopsy; this is post-treatment monitoring, not surveillance of an untreated gland.

## 9. Shared Decisions and the Proposed Pathway

Shared decisions should explain the initial diagnosis, uncertainty from sampling, planned follow-up, triggers for reassessment, treatment morbidity and competing health risks. A review of decision support interventions covered heterogeneous prostate/bladder populations and does not prove improved AS cancer outcomes [51]. In the 327-person Healium trial, both active decision aids reduced decisional conflict at six weeks; the study did not demonstrate an AS-specific survival or adherence benefit [52]. Counseling can support informed participation without treating a preference for delayed care as an anxiety disorder.

**Figure 1:**
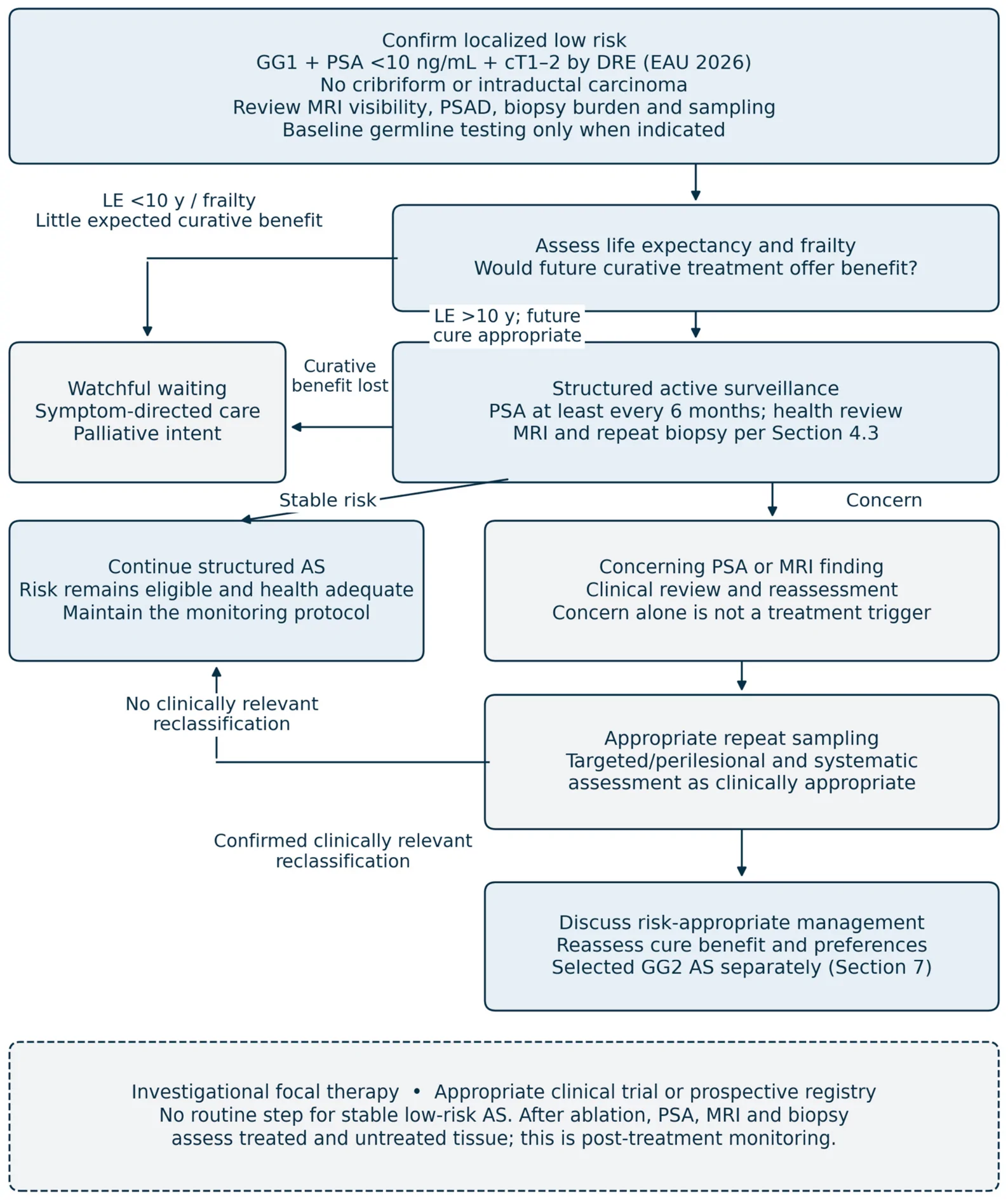
Author-proposed pathway for confirmed low-risk localized prostate cancer; not prospectively validated. Low risk follows the stated EAU 2026 classification (GG1, PSA < 10 ng/mL, cT1–2 by DRE); historical cohorts often used narrower stage/volume definitions. MRI visibility, PSAD and tumor burden refine baseline assessment. LE denotes estimated life expectancy; frailty and expected curative benefit qualify the approximate ten-year boundary. Concerning PSA/MRI findings prompt reassessment and appropriate sampling. Confirmed clinically relevant reclassification prompts a risk-specific discussion; selected GG2 AS is considered separately in Section 7. The focal treatment note is investigational and outside the routine sequence. Follow-up details and conditional biopsy exceptions are specified in Section 4.3. Sources: EAU 2026, imaging evidence and contemporary surveillance outcomes [1,4,26,27,29].

Figure 1 is an author-proposed synthesis and has not been prospectively validated. Baseline low-risk confirmation includes grade, PSA, DRE stage, MRI visibility, PSAD, tumor burden and exclusion of adverse histology. Germline testing is assessed once when indicated, not used as a changing per-visit trigger. Health assessment then separates structured AS from WW. Stable risk leads to continued AS; concerning PSA/MRI findings lead to clinical review and appropriate repeat sampling; confirmed relevant reclassification leads to a risk-specific treatment discussion. If no relevant reclassification is found, AS continues. Changing health can redirect AS to WW as an explicit change in intent. The investigational focal therapy note is outside this routine sequence. Table 5 summarizes management choices according to risk, treatment intent and evidentiary status.

## 10. Future Directions and GG1 Terminology

The debate over whether GG1 should retain the cancer label reflects its indolent biology and the harms of diagnosis. However, biopsy-detected GG1 is a sampling result, not proof that the entire prostate contains no higher-grade focus. Language should communicate low current risk without erasing the need to confirm eligibility and monitor appropriately [4,11]. The practical priority is reducing avoidable treatment while maintaining reliable detection of clinically important disease.

Future work should evaluate the incremental value of biomarkers beyond MRI, PSAD and pathology in independent cohorts and then test management outcomes prospectively. G-MAJOR and HIFUSA address different investigational questions; their registered objectives cannot be substituted for results [33,50]. EAU notes limited small/short-term PSMA-PET evidence for AS selection and does not recommend its use outside trials for that purpose [4]. Useful research endpoints include missed clinically significant reclassification, metastasis, patient-reported outcomes, access, treatment burden and economic performance in the health systems where a pathway will operate.

## 11. Limitations

This narrative review uses purposive selection without exhaustive or independent duplicate screening or formal risk-of-bias scoring. Complete strings, counts and filter settings for the initial search are not documented; the reproducible supplementary search is reported separately. Abstract-only access limits the comparison of NCCN/AUA 2026 recommendations and selected study methods. Retrieved counts are not screened- or included-study counts. Historical trials and surveillance cohorts differ in risk mix, diagnostic era, monitoring, endpoint definitions and follow-up. Low late-event counts, selective biopsy verification, tissue-profiling attrition, competing mortality and residual confounding limit precision and comparability. Economic findings depend on assumptions and local costs. The proposed pathway is an interpretive synthesis, not a validated decision rule, and selected GG2 or post-ablation evidence is not equivalent to untreated low-risk AS.

## 12. Conclusions

Consistent with the objective stated in the Introduction, the evidence reviewed here indicates that structured AS is the preferred initial management for most suitable men with confirmed low-risk localized prostate cancer who retain the prospect of benefit from curative treatment. Historical WW, observation and ProtecT active monitoring should be interpreted within their own eras and populations. Established components are careful clinical/pathological selection, exclusion of cribriform/intraductal histology, MRI-informed sampling, planned reassessment and decisions that account for life expectancy and competing health risks. Stable risk supports continued AS; concerning PSA/MRI findings require reassessment before clinically meaningful reclassification is accepted. Transition to WW may become appropriate as curative benefit declines.

Selected tissue classifiers and gene-specific hereditary information may inform uncertain decisions, but heterogeneous associations and negative findings do not establish improved outcomes from molecularly guided surveillance. MRI-based economic strategies remain conditional model findings, psychological outcomes vary and focal therapy remains investigational in this population. The proposed pathway therefore distinguishes routine AS from selected adjuncts and research, and makes the boundary between suspicion, confirmation and treatment explicit.

## Figures and Tables

**Table 1:** Review design, retrieval and appraisal.

Component	Documented Approach and Boundary
Review identity	Historical, evidence-based narrative synthesis. No systematic review registration, PRISMA flow diagram or quantitative pooling.
Initial retrieval	PubMed/MEDLINE, Scopus and reference checking up to 10 September 2026. The documented summary does not retain exact strings, counts or filter settings.
Initial selection	Purposive selection for explanatory value by three reviewers, with disagreements resolved through discussion; no independent duplicate screening.
Revision retrieval	PubMed broad query plus six topic queries executed on 10 October 2026; exact strings and overlapping retrieval counts in Appendix A. Targeted primary source verification rather than screening of every hit.
Restrictions	No language or publication type filters in the supplementary queries. Original restrictions cannot be verified.
Question-specific hierarchy	Randomized trials for comparisons; prospective cohorts for absolute AS outcomes; imaging meta-analysis for diagnostic performance; molecular analyses for association/incremental prediction; registries for uptake; models for economics; official guidelines for recommendations.
Appraisal	Design-specific limitations described beside the evidence. No formal risk-of-bias scoring or reconstructed exclusion log.
Access limits	Abstract-only sources are identified where unverified methods or recommendation details would affect interpretation. Trial objectives/status are kept separate from published results.

**Table 2:** Central trials and cohorts with absolute outcomes interpreted in their diagnostic era.

Study and Population	Conservative Protocol and Follow-Up	Absolute Endpoints and Interpretation
SPCG-4 [18] Randomized *n* = 695; clinically detected, risk-mixed, largely prescreening.	WW versus prostatectomy; follow-up up to 29 years.	Prostate cancer deaths 71/347 surgery versus 110/348 WW. Crude event counts; reported RR 0.55 (95% CI 0.41–0.74). Not contemporary low-risk AS.
PIVOT [2] Randomized *n* = 731; early PSA era, risk-mixed.	Observation versus prostatectomy; maximum 22.1 years; survivor median 18.6 years.	All-cause mortality 68% versus 73% for surgery versus observation; absolute difference 5.7 points (95% CI −0.89 to 12). Endpoint is not disease-specific mortality.
ProtecT [3] Randomized *n* = 1643; PSA-detected, >one-third intermediate/high risk on updated characterization.	PSA-triggered active monitoring versus surgery or radiotherapy; median 15 years.	Deaths from prostate cancer: 17/545, 12/553, 16/545; metastases: 51/545, 26/553, 27/545 in monitoring/surgery/radiotherapy order. Monitoring is not MRI-informed AS.
PRIAS [5] Prospective *n* = 2494; Gleason ≤6, T1/T2, PSA ≤10, ≤2 positive cores, PSAD < 0.2.	PSA every 3–6 months; biopsy years 1, 4, 7; median 1.6 years.	415/1480 rebiopsied men reclassified; two-year treatment-free survival 77.3%; disease-specific survival 100% at short follow-up.
Toronto [20] Prospective *n* = 993; low and favorable intermediate risk.	Serial PSA and biopsy; survivor median follow-up 6.4 years, maximum 19.8.	10-/15-year cancer-specific survival 98.1%/94.3%; 15 prostate cancer deaths and 2.8% metastatic events during observation. Risk mix differs from strict GG1 cohorts.
Canary PASS [1] Prospective multicenter *n* = 2155; 90% GG1.	Protocol-directed testing and repeat biopsy; median 7.2 years.	10-year metastasis 1.4% (95% CI 0.7–2.0), prostate cancer death 0.1% (0–0.4), reclassification 43% (40–45), treatment 49% (47–52). 21 metastases/3 disease-specific deaths observed.
Benheddi et al. [16] Single-center *n* = 354, GG1 after MRI plus systematic/targeted sampling.	MRI-era AS; median 6.3 years (IQR 3.2–9.1).	Two metastases and no prostate cancer deaths reported. Ten-year numbers at risk unavailable in accessible abstract; late cumulative estimates are not reproduced.
Englman et al. [29] MRI-led cohort *n* = 1150; 36% GG2, PSA < 20, ≥2 MRIs.	Biopsy triggered by MRI/PSAD; median 64 months (IQR 32–107).	5-year event-free survival for GG1: 91% (95% CI 88–94) MRI-invisible versus 71% (65–78) visible. Composite is upgrade to ≥4+3 or treatment, not metastasis-free survival.

RR, relative risk; IQR, interquartile range. Absolute event counts and time-to-event estimates are labeled separately. Conservative protocols, entry criteria and eras differ; no pooled or causal comparison between cohorts is intended.

**Table 3:** Current guideline comparison with source-access boundaries.

Topic	EAU 2026 Full Guidance	NCCN 5.2026 Published Insights	AUA/ASTRO 2026 Amendment
Selection	GG1, PSA < 10, cT1–2 by DRE in current risk classification; AS for suitable low-risk men. No cribriform/intraductal histology. Selected GG2 separate.	Abstract verifies elimination of the very-low-risk group. Full updated eligibility/AS criteria were not accessible.	Abstract verifies a 2026 amendment; detailed eligibility statements were not accessible.
Confirmatory biopsy	MRI before confirmation if not initially performed (strong); upfront MRI with targeted/perilesional sampling may permit omission (weak).	Updated AS principles announced; timing/conditions not verifiable from abstract.	Detailed timing/conditions not verifiable from accessible abstract.
PSA and MRI	PSA at least six-monthly; no universal fixed MRI interval. MRI plus biopsy for PSA doubling time < 3 years (strong).	Updated AS principles announced; PSA/MRI intervals not verifiable.	Revised imaging guidance announced; exact surveillance intervals not verifiable.
Repeat biopsy	Every 2–3 years until LE < 10 (strong). Selected stable MRI/PRECISE 3 plus stable PSAD < 0.15 may permit omission (weak).	Repeat-biopsy schedule/exception not verifiable from abstract.	Repeat-biopsy schedule/exception not verifiable from abstract.
Tissue biomarkers	Promising selected adjuncts; insufficient evidence to replace conventional assessment routinely.	Detailed 2026 biomarker recommendations not accessible.	Detailed 2026 biomarker recommendations not accessible.
Germline testing	Personal/family/tumor indications with counseling; BRCA2 is not automatic AS exclusion with favorable tumor factors.	Detailed 2026 testing recommendations not accessible.	Detailed 2026 testing recommendations not accessible.
Focal treatment	HIFU/cryo in prospective registry; other modalities in designed trials. Investigational low-risk role.	Abstract specifically urges caution for newly diagnosed patients.	Detailed 2026 focal therapy statement not accessible.

Sources: official EAU 2026 full guideline [4], Spratt et al. [7], Eastham et al. [12]; accessed 10 October 2026. This is a deliberately incomplete comparison where full texts were unavailable. Older NCCN/AUA schedules are not represented as checked 2026 recommendations. Recommendation strengths are stated only when verified.

**Table 4:** Molecular and hereditary evidence with the endpoint and clinical boundary stated explicitly.

Study or Tool	Analyzed Population and Endpoint	Result, Adjustment and Limitation
Decipher 1.0/1.1 [13]	605 AS men within *n* = 1579; major upgrade to GG ≥ 3.	High v1.1 versus low: HR 2.11 (95% CI 1.14–3.89). Follow-up, adjustment and direct performance difference unavailable in abstract; version superiority/utility not established here.
Commercial GPS in PASS [30]	*n* = 432; median 4.6 years; 167 upgrades; 52 adverse-pathology events among 101 prostatectomies.	No upgrade association (*p* = 0.48); adverse-pathology association nonsignificant with PSAD plus grade (*p* = 0.066); no significant added stratification.
PAM50 in MAST [31]	205 enrolled; 159 profiled; 128 baseline profiles (46 Luminal A, 26 Luminal B, 56 Basal); grade progression over up to 3 years.	Unadjusted Luminal B progression-free median 1.7 versus 2.9 years; no significant adjusted association. Nonprogressor median follow-up 1.86 years. Internal, missing-tissue and industry-linked analysis.
MAST Decipher/dGPS/dCCP [32]	141/205 men; 324 GG1 cores, 235 biopsy sessions; coexisting higher grade.	All-core dGPS signal for GG ≥ 3 (nine coexisting cores); none for GG ≥ 2. No highest-volume-core group difference or independent adjusted association. Derived GPS/CCP are research signatures, not interchangeable commercial assays.
Carter carrier cohorts [34]	*n* = 1211; 26 ATM/BRCA1/2 carriers, 11 BRCA2; median 3 years; biopsy reclassification.	Adjusted HR 1.96 (95% CI 1.004–3.84) pooled three-gene set; BRCA2 HR 2.74 (1.26–5.96). Competing-risk adjustment; few carriers and surrogate endpoint.
Brady/PASS carrier analysis [35]	*n* = 437; eight BRCA1/2/ATM carriers, only three BRCA2; median 7.2 years.	No observed reclassification association; broader repair-gene adverse-characteristic HR 0.73 (95% CI 0.29–1.86) adjusted for clinical factors. Wide uncertainty; distinct endpoint.
G-MAJOR [33]	Cluster-crossover randomized study; actual *n* = 805; two-year AS without treatment.	Active, not recruiting; estimated primary completion March 2028. No posted results at access on 10 October 2026; clinical utility remains unreported.
PATROL [37]	Prospective screening of unaffected carriers; primary PSA/MRI positive predictive value.	AS follow-up is secondary. Protocol objectives are not published AS outcome evidence.

AS, active surveillance; CCP, cell-cycle progression; GPS, Genomic Prostate Score; PAM50, 50-gene expression subtype classifier. Model-derived association is not equivalent to improved test-guided patient outcomes.

**Table 5:** Management choices according to risk, treatment intent and evidentiary status.

Option	Appropriate Context	Monitoring and Decision Boundary
Structured AS	Confirmed low risk; adequate life expectancy and eligibility for future cure. Preferred initial strategy for most suitable men [1,4].	PSA, health/clinical review, MRI-informed assessment and repeat biopsy. Stable risk continues AS; concern triggers reassessment, not automatic treatment. Selected EAU biopsy exceptions require context.
WW	Limited life expectancy, substantial frailty or little expected curative benefit [4].	Symptom-directed management. May follow AS when health changes; this represents a change in therapeutic intent rather than nonadherence.
Risk-appropriate radical treatment	Clinically meaningful confirmed reclassification, or an informed choice after reassessing benefits and harms [3,19].	Discuss prostatectomy/radiotherapy according to revised risk and health. PSA/MRI concern requires appropriate confirmation before being treated as progression.
Selected GG2 AS	Separate intermediate-risk exception; limited pattern 4/extent and no cribriform or intraductal carcinoma [4].	Explicitly discuss higher progression risk; strict selection and monitoring. This selected intermediate-risk group is outside the principal low-risk pathway shown in Figure 1.
Focal ablation	Investigational in this low-risk context; prospective trial/registry as appropriate to modality [4,50].	No routine route from stable AS. Post-ablation follow-up evaluates treated and untreated tissue.
Patient support	Understanding, measured distress, preferences and access barriers [40,41].	Distinguish anxiety from patient/physician concern, elective treatment and loss to follow-up. No assumed uniform psychological discontinuation rate.
MRI-based economic strategies	Potentially favorable within selected US model assumptions [14,15].	Modeled thresholds/intervals are not universally validated clinical rules; economic value does not establish biopsy omission safety.

## Data Availability

No new data were created or analyzed in this study. Data sharing is not applicable to this article.

## Supplementary Materials

The following supporting information can be downloaded at: https://www.mdpi.com/article/10.3390/jcm15218290/s1, Supplementary Material S1, completed SANRA self-assessment based on Baethge et al. [17].

## Abbreviations

AS, active surveillance; CCP, cell-cycle progression; DRE, digital rectal examination; GG, grade group; GPS, Genomic Prostate Score; HIFU, high-intensity focused ultrasound; HR, hazard ratio; IIEF-5, International Index of Erectile Function 5; IRE, irreversible electroporation; LE, life expectancy; MAST, Miami Active Surveillance Trial; MRI, magnetic resonance imaging; PAM50, 50-gene expression subtype classifier; PASS, Prostate Active Surveillance Study; PI-RADS, Prostate Imaging Reporting and Data System; PRECISE, Prostate Cancer Radiological Estimation of Change in Sequential Evaluation; PSA, prostate-specific antigen; PSAD, PSA density; QALY, quality-adjusted life-year; VUS, variant of uncertain significance; WW, watchful waiting.

## Appendix A. Executed Supplementary PubMed Strategies

All searches below were submitted on 10 October 2026 using PubMed ESearch with retmax = 0. No language or publication type filters were applied; the date restriction concerns publication date. Counts overlap and were recorded at execution. Queries were used to document supplementary retrieval and support targeted checks, not to claim exhaustive screening. The original PubMed/Scopus searches through 10 September 2026 are not reconstructed by these strings. The publication-date range is 1 January 1900 to 10 October 2026; the YYYY/MM/DD tokens and search-field syntax are retained verbatim within the submitted queries.

### Appendix A.1. Broad Conservative Management Query

Recorded retrieval count: 5030. Submitted query: (“Prostatic Neoplasms”[MeSH Terms] OR “prostate cancer”[Title/Abstract]) AND (“active surveillance”[Title/Abstract] OR “watchful waiting”[Title/Abstract] OR “active monitoring”[Title/Abstract]) AND (“1900/01/01”[Date - Publication] : “2026/10/10”[Date - Publication])

### Appendix A.2. Imaging

Recorded retrieval count: 1255. Submitted query: (“Prostatic Neoplasms”[MeSH Terms] OR “prostate cancer”[Title/Abstract]) AND (“active surveillance”[Title/Abstract]) AND (“magnetic resonance imaging”[Title/Abstract] OR MRI[Title/Abstract] OR PRECISE[Title/Abstract] OR “targeted biopsy”[Title/Abstract]) AND (“1900/01/01”[Date - Publication] : “2026/10/10”[Date - Publication])

### Appendix A.3. Molecular Classifiers

Recorded retrieval count: 91. Submitted query: (“Prostatic Neoplasms”[MeSH Terms] OR “prostate cancer”[Title/Abstract]) AND (“active surveillance”[Title/Abstract]) AND (Decipher[Title/Abstract] OR “genomic prostate score”[Title/Abstract] OR Prolaris[Title/Abstract] OR PAM50[Title/Abstract]) AND (“1900/01/01”[Date - Publication] : “2026/10/10”[Date - Publication])

### Appendix A.4. Germline Findings

Recorded retrieval count: 48. Submitted query: (“Prostatic Neoplasms”[MeSH Terms] OR “prostate cancer”[Title/Abstract]) AND (“active surveillance”[Title/Abstract]) AND (germline[Title/Abstract] OR BRCA2[Title/Abstract] OR ATM[Title/Abstract] OR PATROL[Title/Abstract]) AND (“1900/01/01”[Date - Publication] : “2026/10/10”[Date - Publication])

### Appendix A.5. Economics

Recorded retrieval count: 66. Submitted query: (“Prostatic Neoplasms”[MeSH Terms] OR “prostate cancer”[Title/Abstract]) AND (“active surveillance”[Title/Abstract]) AND (“cost-effectiveness”[Title/Abstract] OR microsimulation[Title/Abstract]) AND (“1900/01/01”[Date - Publication] : “2026/10/10”[Date - Publication])

### Appendix A.6. Psychology and Access

Recorded retrieval count: 286. Submitted query: (“Prostatic Neoplasms”[MeSH Terms] OR “prostate cancer”[Title/Abstract]) AND (“active surveillance”[Title/Abstract]) AND (psychological[Title/Abstract] OR anxiety[Title/Abstract] OR disparities[Title/Abstract] OR AQUA[Title/Abstract]) AND (“1900/01/01”[Date - Publication] : “2026/10/10”[Date - Publication])

### Appendix A.7. Focal Treatment

Recorded retrieval count: 1893. Submitted query: (“Prostatic Neoplasms”[MeSH Terms] OR “prostate cancer”[Title/Abstract]) AND (“focal therapy”[Title/Abstract] OR HIFU[Title/Abstract] OR “partial ablation”[Title/Abstract] OR “vascular-targeted photodynamic therapy”[Title/Abstract]) AND (“1900/01/01”[Date - Publication] : “2026/10/10”[Date - Publication])

## Appendix B. Source-Access Record

Access level refers to the source material available for this revision, not a formal quality rating. Abstract-derived results are kept within the data reported there; absence of detailed methods is not filled by inference. The Moore/Parker record is used only to acknowledge an earlier evolutionary perspective, not to support clinical effects.

**Table A1:** Verification material by source.

Material Accessed	References and Verification Boundary
Official full guidance	EAU 2026 full guideline and classification, diagnostic and treatment chapters [4]; ACMG/AMP sequence-variant interpretation guidance [36]. Relevant recommendations were checked.
Full article text	Yerram, PAM50/MAST, companion MAST, Carter germline, Brady/PASS, Kang, Magnani, PATROL, SOGUG, HAROW, focal cryotherapy, Healium, AQUA, SANRA and Zattoni review [6,11,14,15,17,26,31,32,34,35,37,38,42,48,52]. Relevant methods, endpoints and limitations were consulted. Kang’s accessible article identifies correction of the earlier online version on 25 August 2021.
Indexed abstract and metadata	PASS outcomes, PIVOT, ProtecT outcomes, PRIAS, NCCN Insights, Lowenstein review, Klotz review, AUA amendment, Decipher versions, Benheddi, SPCG-4, ProtecT patient outcomes, Toronto, Ontario, MRI-led cohort, Tzeng, SEER–Medicare, both ASIST reports, serial-MRI meta-analysis, PRECISE v2, PASS GPS, Patel, psychological review, treatment-change review, Finnish PRIAS, PCM301, HIFI, PART, IRE, sexual-function review and decision-support review [1,2,3,5,7,9,10,12,13,16,18,19,20,21,22,23,24,25,27,28,29,30,39,40,41,43,44,45,46,47,49,51]. Full methods/recommendations were not comprehensively accessible; unreported details are withheld.
Bibliographic metadata only	Moore and Parker’s earlier evolutionary perspective [8]. No abstract/full-text content used to support an outcome claim.
Official trial registration	G-MAJOR and HIFUSA registry records [33,50]; status, enrollment, endpoints and estimated dates checked on 10 October 2026. No results were posted.
