# Supplementary material for: From Radical Treatment to Structured Active Surveillance: A Narrative Review of Low-Risk Localized Prostate Cancer
Source: J Clin Med. 2026 Oct 24;15(21):8290. doi: 10.3390/jcm15218290 (PMC13764061; doi:10.3390/jcm15218290)
Supplement: Supplementary file 1 [file jcm-15-08290-s001.zip › jcm-4637354-supplementary.pdf]

SANRA assessment of the revised narrative review

Manuscript jcm-4637354 | Self-assessment of the revised text

This completed assessment uses SANRA's six items, each scored 0–2. It evaluates the quality of narrative-review presentation and reasoning; it is not a reporting checklist for a systematic review, a risk-of-bias tool or independent certification. The total self-assessment score is 11/12. The search item is deliberately limited because the new documented search cannot reconstruct the original procedure.

SANRA item	Score	Basis in the revised manuscript
1 Importance to readers	2/2	Section 1 and Abstract explain overtreatment, the opportunity for cure and the consequences of confusing WW, active monitoring and AS. The review connects those distinctions to decisions for localized low-risk disease.
2 Specific aims	2/2	Section 1, paragraph beginning "Evolutionary accounts and surveillance frameworks already exist…" states the historical/evidentiary contribution and the aim to distinguish routine AS, selected adjuncts and research.
3 Search description	1/2	Methods 2.1–2.2, Table 1 and Appendix A describe the initial databases, date and three-reviewer purposive selection, and separately report executed October PubMed queries with exact strings, filters and overlapping counts. Complete initial search strings, counts and filter settings are not documented; no exhaustive screening is claimed.
4 Referencing	2/2	Clinical recommendations and disputed claims are tied to primary studies, current verified guidance or trial registrations. Bibliographic metadata/DOIs were checked. Inaccessible recommendation details are not attributed to older guideline versions.
5 Scientific reasoning	2/2	Methods 2.3 sets question-specific evidence priorities. Sections 3–8 address selection, competing risk, mixed-grade populations, MRI verification, confounding, profiling attrition, negative studies and model assumptions. Association, incremental prediction and clinical utility are separated.
6 Relevant endpoint data	2/2	Table 2 gives populations, protocols, follow-up and absolute outcomes. Sections 4–5/Table 3 specify endpoints, denominators and uncertainty; unavailable follow-up/at-risk details are withheld. Biopsy outcomes, treatment change, metastasis and death are not treated as equivalent.

Total 11/12

Assessment boundary: this score is a self-assessment of the revised text, not independent certification. A maximum score is not assigned solely because supplementary queries have been added; the original search documentation remains incomplete. Items 4–6 do not certify every source as full-text verified, and the manuscript explicitly identifies abstract-only access limits.

Source: Baethge C, Goldbeck-Wood S, Mertens S. SANRA—a scale for the quality assessment of narrative review articles. Research Integrity and Peer Review. 2019;4:5. https://doi.org/10.1186/s41073-019-0064-8. Item headings above are concise paraphrases of the six published criteria.
